# Garnet peridotites reveal spatial and temporal changes in the oxidation potential of subduction

**DOI:** 10.1038/s41598-018-34669-0

**Published:** 2018-11-06

**Authors:** Andrea Rielli, Andrew G. Tomkins, Oliver Nebel, Joël Brugger, Barbara Etschmann, David Paterson

**Affiliations:** 1 Dipartimento di Scienze della Terra, Università di Pisa, Via S. Maria 53, 56126 Pisa, Italy; 2 School of Earth, Atmosphere and the Environment, Monash University, Melbourne, Victoria, 3800 Australia; 3 Australian Synchrotron, 800 Blackburn Road, Clayton, Victoria, 3168 Australia

## Abstract

Changes in the oxygen fugacity (*f*O_2_) of the Earth’s mantle have been proposed to control the spatial and temporal distribution of arc-related ore deposits, and possibly reflect the evolution of the atmosphere over billions of years. Thermodynamic calculations and natural evidence indicate that fluids released from subducting slabs can oxidise the mantle, but whether their oxidation potential varied in space and time remains controversial. Here, we use garnet peridotites from western Norway to show that there is a linear decrease in maximum *f*O_2_ with increasing depth in the mantle wedge. We ascribe this relation to changes in the speciation of sulfur released in slab fluids, with sulfate, controlling maximum oxidation, preferentially released at shallow depths. Even though the amount of sulfate in the Precambrian oceans, and thus in subducted lithologies, is thought to have been dramatically lower than during the Phanerozoic, garnet peridotites metasomatised during these two periods have a comparable *f*O_2_ range. This opens to the possibility that an oxidised mantle with *f*O_2_ similar to modern-day values has existed since the Proterozoic and possibly earlier. Consequently, early magmas derived from partial melting of metasomatised mantle may have had suitable *f*O_2_ to generate porphyry Cu-Au and iron-oxide Cu-Au deposits.

## Introduction

**Figure 1:**
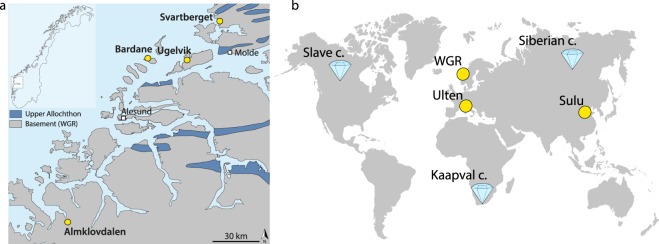
Samples localities. (**a**) Schematic map of the peridotite localities in the WGR of Norway considered in this study, modified from ref.^17^. (**b**) Location of other garnet peridotites considered from the literature. Diamonds represent kimberlite localities whereas yellow spots are orogenic garnet peridotites.

There has been a long running debate as to whether arc magmas inherit their elevated oxidation states from the mantle or acquire them during migration through the crust^1,2^. Since the onset of plate tectonics, subduction has introduced surficial material into the Earth’s mantle, some of which had the potential to modify its oxidation state^3,4^. It has been shown that fluids released through dehydration of subducting slabs may promote metasomatic oxidation of the overlying mantle wedge, by introducing oxidised carbon and sulfur^5–7^. Altered oceanic crust provides the greatest input of sulfur in subduction zones^6^, and the abundance of oxidised sulfur released from it is thought to vary as function of pressure and temperature^8^, potentially leading to changes in the oxidation potential of slab fluids with depth. The amount of oxidised species in subducted lithologies may have also changed during the Earth’s history. Due to the paucity of dissolved sulfate in seawater prior to the second great oxidation event^7,9^, the redox budget of early subduction zones may have been lower than during the Phanerozoic^7^. To investigate changes in mantle wedge *f*O_2_ as a function of depth we examined thirty-six new samples that we collected from four well-studied peridotite localities in the Western Gneiss Region (WGR) of Norway: Ugelvik, Bardane, Svartberget and Almklovdalen (Fig. 1a). These peridotites were initially modified by high degrees of partial melting in the Archean^10,11^, and record variable intensities of subsequent subduction-related metasomatic alteration at different times and depths (see below). Because these peridotites were mechanically emplaced into subducting continental crust they derive from the near-bottom end of the mantle wedge^12^. Based on previously published *f*O_2_ data for the Ugelvik and Bardane peridotites^13,14^, we selected five new samples to obtain the widest range of depths possible.

## Geological Background

**Figure 2:**
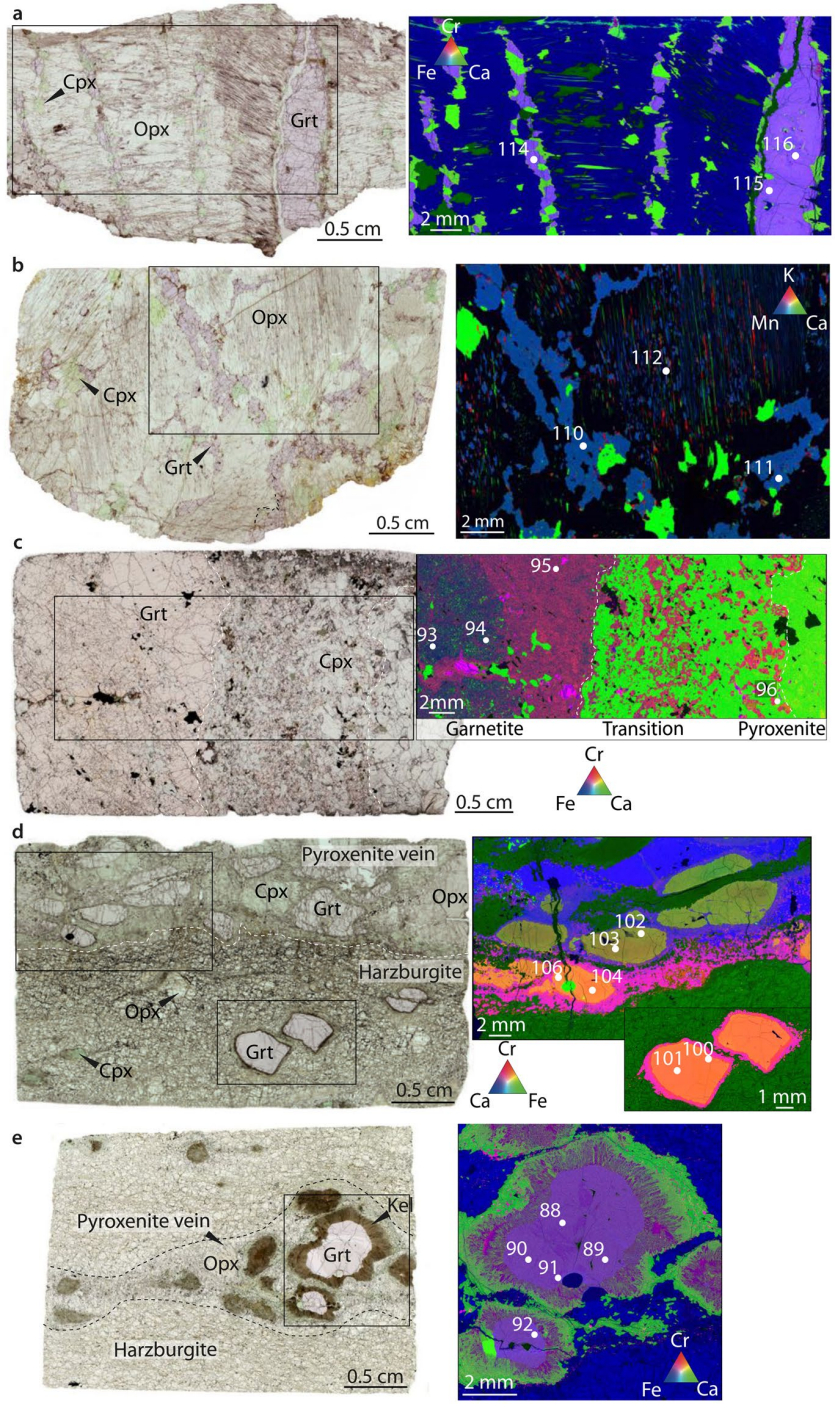
Examples of orogenic peridotite samples from the WGR of Norway considered in this study. On the left is reported the scanned thin section, and on the right the compositional maps of key areas where the XANES spectra were collected (white spots). The numbers associated with the white spots identify the spectra in in Table 1 and Supplementary Table S1 (**a**) UGL03; (**b**) BDN02; (**c**) SVT06; (**d**) GSD08; (**e**) GSD07.

The Ugelvik peridotites have extremely depleted trace element compositions, implying that they escaped alteration by slab fluids^11^. From this locality we studied a garnet pyroxenite (UGL03; Fig. 2a). The proportion of the majorite component in garnet in this sample indicates equilibration at ~7.3 GPa (Methods). Metasomatism by slab fluids at ~500 Ma (ref.^15^) has been identified in the Bardane peridotite on the base of : i) strong enrichment in light-rare earth elements (LREE) and large-ion lithophile elements (LILE), such as Ba, Sr, Rb, U and Th; ii) elevated ^87^Sr/^86^Sr and low ^143^Nd/^144^Nd (ref.^15,16^); and iii) sulfur isotope signature compatible with fluid produced by dehydration of subducting slabs^17^. These fluids produced different assemblages; at ~3 GPa (M3-1 and M3-2 stages) and at ~6 GPa (M3-3 stage^14^). From Bardane we studied sample BDN02 (Fig. 2b), a phlogopite- and carbonate-bearing garnet pyroxenite recording the M3-1 event. The Svartberget peridotite experienced alteration by felsic slab-derived material at ~397 Ma that produced decimeter to meter wide veins with garnetite cores and pyroxenite margins strongly enriched in LILE and LREE, recording pressure of ~5.5 GPa (ref.^18^). SVT06 samples the transition between vein core and margin (Fig. 2c). The Almklovdalen peridotite was metasomatised by basaltic melt at ~1.0 Ga (ref.^19^) and the melt is preserved as pyroxenite veins formed at ~3.5 GPa (ref.^20^). The derivation of this metasomatic agent from a subducted slab is suggested by the sulfur isotopic composition of sulfides hosted in metasomatised domains^17^. From this locality we considered GSD08 (Fig. 2d), sampling the transition between garnet pyroxenite and metasomatised harzburgite, and GSD07, a garnet pyroxenite (Fig. 2e).

## Results and Discussion

**Figure 3:**
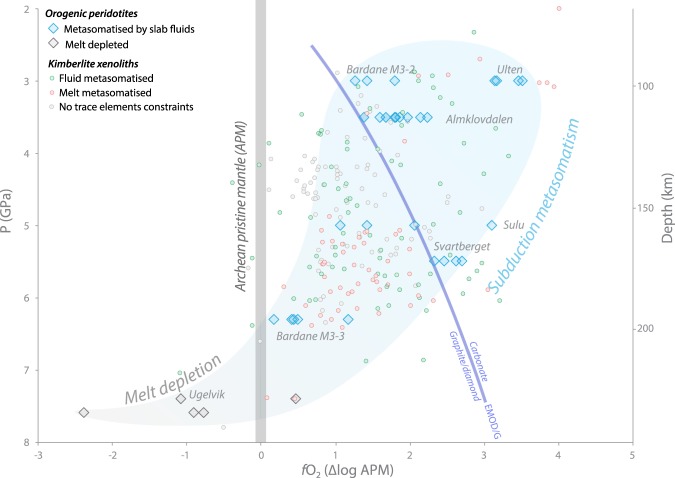
*f*O_2_-depth relation in orogenic peridotites and kimberlite xenoliths. Blue diamonds represent mantle metasomatised by Phanerozoic subductions, new data from this work is integrated with values from ref.^13^, the Ulten amphibole-bearing garnet peridotite, the magnesite-phlogopite garnet peridotite from Sulu^22^, and a garnet-websterite from Bardane^14^. The latter, unlike our samples, records the M3-3 metasomatic event in veins crosscutting the earlier assemblages. The spots represent the *f*O_2_ of peridotite xenoliths recovered from kimberlite magmas^23–30^. Blue spots are xenoliths with sinusoidal trace elements patterns in garnet, indicating metasomatism by fluids^48^, whereas red spots are xenoliths with garnet showing normal patterns, suggesting alteration by melts^48^. Grey spots are xenoliths without trace elements constrains. For the sake of comparison the *f*O_2_ of all samples was recalculated using the oxybarometric calibration of ref.^21^ and normalised to the modelled Archean pristine mantle *f*O_2_ (see Methods).

The *f*O_2_ of selected samples was calculated by applying garnet oxybarometry^21^, using the Fe^3+^/Fe_tot_ of garnets measured by synchrotron Fe-edge X-ray absorption near edge structure spectroscopy (XANES; Methods). Results are reported in Table 1 and graphically in Fig. 3, together with literature data for the Bardane and Ugelvik peridotites^13,14^ and other garnet peridotites metasomatised by subduction at similar depths during the Phanerozoic^22^. In Fig. 3 for comparison we have also reported the *f*O_2_ of cratonic mantle xenoliths brought to the surface by kimberlite magmas from the Siberian, Kaapvaal and Slave cratons^23–30^ (Fig. 1b). Because the calculated *f*O_2_ of a garnet peridotite with fixed composition decreases with increasing pressure^31^, depth-related changes in oxidation state due to metasomatism can be obscured. One way to overcome this issue is to normalise the results to the *f*O_2_ of an unmetasomatised mantle end-member.

**Table 1:** *f*O_2_ results for the Norwegian orogenic peridotites considered in this study. The P, T and Fe^3^^+^/Fe_tot_ used for *f*O_2_ calculation are also reported.

Sample	Grt spot	Spectrum n°	Fe^3+^/Fe_tot_	P (Gpa)	T (C°)	*f*O_2_ (ΔlogFMQ)	*f*O_2_ (ΔlogAPM)
GSD07_1	core	88	0.038	3.5	850	−0.9	1.7
GSD07_2	core	89	0.033	3.5	850	−1.2	1.4
GSD07_3	core	90	0.034	3.5	850	−1.2	1.4
GSD07_4	core	91	0.051	3.5	850	−0.5	2.1
GSD07_5	core	92	0.046	3.5	850	−0.6	2.0
GSD08_1	core	100	0.042	3.5	850	−1.0	1.6
GSD08_2	core	101	0.036	3.5	850	−0.8	1.8
GSD08_3	core	102	0.044	3.5	850	−0.7	1.8
GSD08_4	core	103	0.042	3.5	850	−0.7	1.9
GSD08_5	core	104	0.039	3.5	850	−0.9	1.7
GSD08_6	core	106	0.054	3.5	850	−0.4	2.2
BDN03_1*	rim	124	0.039	6.3	1000	−3.5	0.2
BDN03_2*	core	125	0.049	6.3	1000	−3.2	0.5
BDN03_3*	core	126	0.046	6.3	1000	−3.2	0.4
BDN02_1	corona	110	0.018	3.0	800	−1.0	1.4
BDN02_2	vein	111	0.030	3.0	800	−1.1	1.3
BDN02_3	exolution	112	0.021	3.0	800	−0.5	1.8
UGL01_1*	core	97	0.014	7.6	1573	−6.4	−2.4
UGL01_2*	core	98	0.038	7.6	1573	−4.8	−0.8
UGL01_3*	rim	99	0.033	7.6	1573	−4.9	−0.9
UGL03_2	vein core	115	0.018	7.4	1539	−5.0	−1.1
UGL03_3	vein core	116	0.058	7.4	1539	−3.5	0.5
SVT06_1	core	93	0.081	5.5	800	−1.9	2.5
SVT06_2	core	94	0.095	5.5	800	−1.7	2.7
SVT06_3	transition	95	0.078	5.5	800	−2.1	2.3
SVT06_4	pyroxenite	96	0.089	5.5	800	−1.8	2.6

Samples with asterisk are from ref.^13^.

### The *f*O_2_ of unmetasomatised mantle

**Figure 4:**
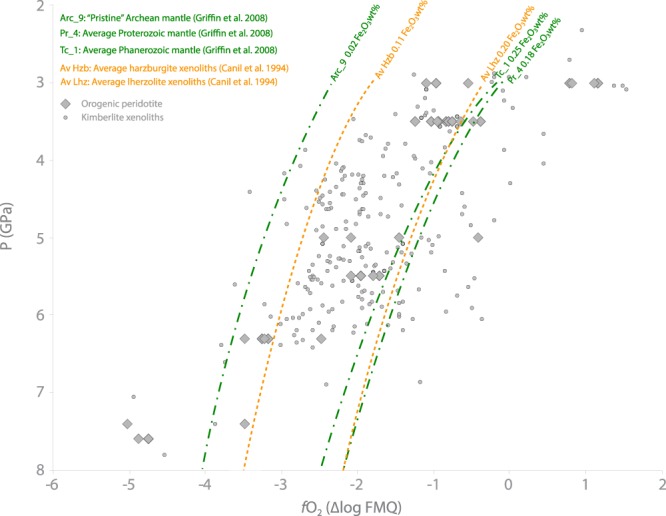
*f*O_2_-depth trends for different mantle end-members. Grey diamonds and spots represent respectively orogenic garnet peridotites and garnet peridotite xenoliths hosted in kimberlites reported in Fig. 3.

It has been shown that the pre-metasomatic end-member of the Norwegian orogenic peridotites is a depleted harzburgite/dunite lithology generated by high-degrees of partial melting in the Archean^10,11,32^. A similar depleted end-member has also been suggested to represent the pristine unmetasomatised mantle that underlies Archean cratons worldwide^33^. By comparison, in areas where the continental crust is younger, the underlying mantle has largely been refertilised during major tectonic events, in some cases involving multiple subduction cycles^33^. These mantle end-members have been modelled by ref.^33^ by combining data from the orogenic peridotites of Norway and kimberlite xenoliths worldwide. They propose an Archean pristine mantle (APM) composition, represented by depleted dunite/harzburgite (Arc_9), and refertilised end-member compositions for Proterozoic (Pr_4) and Phanerozoic (Tc_1) mantle. Because of the large dataset of natural samples used in the definition of these end-members^33^ and their validation on geophysical basis^33,34^, we chose these as starting compositions for further *f*O_2_ modelling (Methods) with the aim of identifying the *f*O_2_ of an unmetasomatised mantle end-member. The Archean pristine mantle composition (Arc_9) has the lowest *f*O_2_ of all the considered end-members; ~95% of the considered samples, including both orogenic peridotites and kimberlite-hosted peridotite xenoliths, show higher *f*O_2_ (Fig. 4). Most of our samples, and the other orogenic peridotites for which there are published *f*O_2_ data, have been affected by some degree of metasomatic alteration^13,14,22^. We have also found that, on the basis of trace element concentrations in garnet, all kimberlite xenoliths for which there are relevant data^23,24,28,29^ have been affected by some degree of metasomatic alteration (Fig. 3). This can explain their higher *f*O_2_ compared to the modelled Arc_9 composition (Fig. 4). Only the samples from the Ugelvik peridotite, UGL01 and UGL03, have lower *f*O_2_ than Arc_9. This peridotite have experienced high degrees of partial melting in the Archean^11^ and the trace element concentrations in garnet show distinct steep REE patterns, which confirm its extremely depleted nature and indicate that it has not interacted with metasomatic fluids since the Archean^11^. Thus, the samples from the Ugelvik peridotite may represent the best Archean pristine mantle end-member for our metasomatised samples. Pr_4 and Tc_1 have higher *f*O_2_, averaging ~2 log units above Arc_9 (Fig. 4). Most of the considered samples (83%) have *f*O_2_ between the Arc_9 and Pr_4/Tc_1 *f*O_2_ values, in line with the different extents of metasomatic alteration reported for these samples^14,28,35^. Only the 17% of the samples show higher *f*O_2_ than Pr_4/Tc_1, and these may represent mantle domains altered by particularly oxidising agents. To constrain this calculation, we also modelled *f*O_2_-depth trajectories starting from the average bulk composition of harzburgite and lherzolite xenoliths from ref.^36^, which had Fe_2_O_3_ concentrations directly measured by Mössbauer spectroscopy^36^. With this approach the averaged harzburgite (5 samples), which is representative of depleted mantle, and has 0.09 wt% more Fe_2_O_3_ than Arc_9, plots ~0.3 log units above the Arc_9 curve, which is less than the uncertainty in the *f*O_2_ calculation^21^. The average lherzolite composition (14 samples), representative of fertile mantle, has Fe_2_O_3_ content of 0.20 wt%, giving *f*O_2_ values between Tc_1 and Pr_4 at most pressures. This confirms that Tc_1 and Pr_4 are representative of fertile mantle, whereas Arc_9 best represents the pre-metasomatic Archean pristine mantle (APM) protolith for the Norwegian peridotites and for the cratonic mantle samples.

### A redox profile through subduction zone mantle

In order to graphically visualise changes in oxidation state of mantle samples as a function of depth we normalised the *f*O_2_ of samples reported in Fig. 3 to the *f*O_2_ of the modelled APM. It can be seen that all metasomatised samples have higher *f*O_2_ than APM and define a trend of decreasing *f*O_2_ with increasing depth, down to ~200 km, below which *f*O_2_ values are in the range of unmetasomatised mantle or lower. This suggests that with increasing depth the oxidative potential of subduction decreases, and becomes minimal below ~200 km, effectively creating a zonation in mantle wedge *f*O_2_. The main oxidants in slab fluids are carbon and sulfur^7^, and although melts might preferentially mobilise Fe^3+^ (ref.^37^) its abundant addition to the mantle wedge has been excluded^5,38,39^. Reduction of oxidised carbon (e.g., CO_2_) and sulfur (e.g., SO_4_^2−^ or S_3_^−^) during mantle metasomatism can increase mantle *f*O_2_ via redox reactions^3,6,13^. Carbonate minerals, diamond and graphite are present in samples from western Norway metasomatised at depths between ~100 and ~200 km^13,14,22^, indicating that carbon was mobilised in fluids across a wide range of depths. CO_2_ reduction can increase *f*O_2_ up to carbonate stability, defined by the EMOD/G line (enstatite-magnesite-olivine-diamond/graphite, Fig. 3; ref.^40^). Further oxidation requires reduction of oxidised sulfur. In subducting oceanic crust the dominant sulfate mineral is anhydrite and it is dissolved at relatively shallow depths in fluids released across the blueschist-eclogite transition^8^. Instead, at greater depths sulfur is thought to be released, through conversion of pyrite to pyrrhotite, in the form of H_2_S (ref.^41^) or S_3_^−^ (ref.^42^).

Samples metasomatised at depths ≤160 km have *f*O_2_ above the EMOD/G buffer (Fig. 3), requiring the introduction of oxidised sulfur. In contrast, mantle metasomatised at deeper levels has *f*O_2_ below the EMOD/G buffer, suggesting that only shallower fluids introduced a sufficient amount of oxidised sulfur to exceed the EMOD/G buffer. Barite (BaSO_4_) is the dominant sulfur-bearing mineral in some samples metasomatised at depths ≥160 km, such as those from the Bardane (BDN03; Ref.^13^) and Svartberget (SVT06) peridotite, which intuitively suggests that sulfate was carried in the fluid. However, it has been previously suggested^13^ that because Ba is immobile in presence of SO_4_^2−^ (ref.^43^), it is more likely that S_3_^−^ was the dominant sulfur species in those metasomatic fluids. Oxidation by reduction of S^6+^ in sulfate to S^2−^ to form sulfide allows conversion of 8 moles of Fe^2+^ to Fe^3+^ for each mole of sulfate reduced, whereas 3 moles of S_3_^−^ need to be reduced to oxidise 5 moles of ferrous iron. Therefore, sulfate is a stronger oxidant than S_3_^−^ and the observed decreasing *f*O_2_ with increasing depth is reconcilable with preferential release of S^6+^ at shallower depths, and progressive increase in S_3_^−^/S^6+^ ratio as depth increases. Shallowly released fluids would also progressively remove carbonate by dissolution^44^ leaving deeper fluids comparatively depleted in CO_2_.

The other key influence on the variation in mantle oxidation with depth is the amount of oxidising agent added to a given mantle domain. There is a progressive decrease in the amount of fluid released from subducting slabs as hydrous phases are progressively diminished after a peak in fluid production across the blueschist-eclogite transition^8^, becoming minimal below ~200 km^45^. So, it is anticipated that there should be less fluid-driven metasomatism at deeper levels, although in the Norwegian samples metasomatised from ~100 to ~200 km depth we did not find considerable differences in the amount of H_2_O-bearing phases, or degree of alteration. In addition, fluid/melt with a given redox budget produces stronger, more localised oxidation when focused through narrow zones than when it interacts pervasively with larger mantle domains^6,7^. This variation in volume of fluid and extent of fluid-focusing would produce scatter in the *f*O_2_ of metasomatised mantle, potentially explaining the broad range in *f*O_2_ of the orogenic peridotite samples at any given depth (Fig. 3).

We suggest that the preferential release of different oxidants as a function of depth, in combination with a progressive decrease in the amount of fluid released from the slab, are responsible for the observed zonation in mantle wedge *f*O_2_. These factors provide an explanation for the decreasing *f*O_2_ of arc magmas with increasing distance from the trench observed in different arcs worldwide^46,47^, and related spatial variations in ore deposit types, where Cu-Au porphyries that require higher *f*O_2_ are distributed closer to the trench^8^.

### The oxidation potential of subduction through time

In Fig. 3, we have reported also the *f*O_2_ of garnet peridotite xenoliths brought to the surface by kimberlite magmas from the Siberian, Kaapvaal, and Slave Cratons^23–30^ (Fig. 1b). These are grouped on the basis of the trace element composition of garnet^48^: blue spots represent samples metasomatised by fluids, whereas red spots are samples metasomatised by melts. It can be seen that metasomatism in the cratonic mantle produced an increase in *f*O_2_ comparable to that promoted by subduction fluids during the Phanerozoic. Many of these samples have *f*O_2_ above the EMOD/G line, therefore requiring reduction of oxidised sulfur (Fig. 3). Information regarding sulfate stability at mantle conditions is limited; however, experiments have shown that at 1.5 GPa sulfate becomes stable in basaltic melt at *f*O_2_ ≥ FMQ + 0.5 (ref.^49^), and that the stability of sulfate in aqueous fluids at similar pressures requires even higher *f*O_2_ (ref.^50^). Because the *f*O_2_ of deeper mantle is consistently lower than FMQ (ref.^31^), oxidised sulfur in cratonic mantle is unlikely to be derived from unaltered asthenosphere; instead, the most plausible source is subducted material. The cratonic mantle sampled by kimberlites formed as residue after high degrees of partial melting largely in the Archean and records different events of subsequent refertilisation^33^. Trace element signatures and isotopic composition of metasomatic silicates in kimberlite peridotite xenoliths^51^ and diamonds^52^ indicate that subduction fluids played a major role in the refertilisation of the cratonic mantle. The ages of subduction events that modified these mantle domains can be constrained by considering the ages of eclogite xenoliths recovered from kimberlites. These are thought to represent oceanic crust that was subducted and dehydrated in the sub-continental lithospheric mantle^53,54^. Dating of eclogite xenoliths across the Siberian, Kaapvaal and Slave cratons yielded Mesoarchean to Paleoproterozoic ages^55–57^, and because the amalgamation of these cratons largely ended in the Proterozoic^58^, the possibility that the cratonic mantle has been significantly affected by younger subduction events is unlikely. Therefore, the oxidised sulfur necessary for the oxidation of the cratonic mantle to values above the EMOD/G buffer was likely supplied by ancient, pre-Phanerozoic, subduction events.

This observation suggests that the oxidative potential of subduction may have not changed over billions of years and that similar proportions of oxidised sulfur to that subducted in the Phanerozoic were introduced into the mantle during the Proterozoic and possibly Archean. This is in apparent conflict with the suggestion that dominantly anoxic conditions and minimal sulfate existed in the deep oceans^9^, and thus in subducted lithologies, in the Proterozoic. Alternatively, it may be that sulfate may not need to be present to produce oxidised slab fluids containing sulfate species; sulfate could be produced during slab dewatering by sulfide oxidation via reduction of other redox-sensitive elements. During subduction of serpentinised lithospheric mantle, the Fe^3+^/∑Fe of serpentine minerals decreases, and it has been argued that this reduction of ferric iron promotes sulfide oxidation, but only in the most intensely serpentinised lithosphere, generating sulfate-bearing fluids that migrate into the mantle wedge^38,59^. Importantly, the genesis of oxidised fluids through this process is independent of the oxygenation of the oceans or the atmosphere, because serpentinisation is driven by H_2_O dissociation^60^, and thus would have occurred during the Archean and Proterozoic. However, the altered oceanic crust is thought to be by far the most important fluid contributor to the sub-arc mantle during subduction^6,45^, whereas the lithospheric mantle and sediment veneer are only minor contributors. Perhaps the simplest explanation is that sulfate was present in sufficient abundance in Proterozoic oceans to drive oxidation of the oceanic crust initiating a global redox cycle. The data reported in Fig. 2 suggest that Proterozoic magmas derived from partial melting of metasomatised mantle likely had suitable *f*O_2_ for the genesis of porphyry Cu-Au deposits^37^, and other ore deposits associated with oxidised magmas such as iron-oxide copper gold (IOCG) deposits, and some magmatic sulfide deposits^61^. The predominantly Phanerozoic distribution of porphyry Cu-Au deposits may thus not be related to major changes in mantle wedge *f*O_2_ (ref.^7^), but rather to preservational bias because they form in the upper levels of mountain belts^7,62^. IOCG deposits are relatively abundant in the Proterozoic and may be associated with partial melting of oxidised metasomatised mantle.

## Methods

### Synchrotron XANES spectroscopy and MAIA X-ray fluorescence imaging

Fe K-edge XANES spectroscopy has been used to determine the Fe^3+^/Fe_tot_ ratio in garnets. The experiment was conducted at the Australian Synchrotron X-ray Fluorescence Microscopy (XFM) beamline^63^ using a single element Vortex detector. Multiple XANES spectra were acquired for each garnet to constrain intra-grain variations in Fe^3+^/Fe_tot_. The spots where the spectra were acquired are reported in Fig. 1 and Supplementary Fig. 2. The raw data were normalised using the mback software^64^. The Fe^3+^/Fe_tot_ ratio in garnet was calculated using the technique described by ref.^65^. This is based on the establishment of an empirical calibration curve, which relates the ratio of the intensity at 7138.4 and 71.61.7 eV in the XANES spectra of a series of standard garnets to their Fe^3+^/Fe_tot_ values determined by Mössbauer spectroscopy. The Fe K-edge XANES spectra of garnets in the studied samples were acquired in the same analytical session of those in the samples from Bardane (BDN03) and Ugelvik (UGL01) previously published in ref.^13^, thus the same standards (Supplementary Table S1) and calibration curve (Supplementary Fig. S1) of ref.^13^ have been used for the calculation of the Fe^3+^/Fe_tot_ ratios in this study. Results from the XANES experiment for the unknowns are reported in Supplementary Table S1. The precision of the XANES analyses from standard deviation is 0.0037 and the error for the Mössbauer analysis of the standards is 0.01. Elemental maps were acquired at the Australian Synchrotron XFM beamline. The samples were raster scanned in continuous mode with a monochromatic (18.5 keV) X-ray beam focused to 1.5 × 1.5 µm^2^. A 396-element Maia detector^63,66^ collected the X-ray fluorescence and scatter emitted with 1.9 millisec/pixel dwell and the data were processed using GeoPIXE II (ref.^66^).

### Oxygen fugacity calculation and modelling

Oxygen fugacity was calculated using the *GtfO2* software from ref.^21^. This software incorporates four independent oxybarometers and yields optimum log *f*O_2_ estimates by the method of least squares. The uncertainty reported in calculating the *f*O_2_ with this approach ranges from 0.6 to 0.9 log units^21^. The compositions of garnet, olivine and orthopyroxene used for the *f*O_2_ calculations are reported in Supplementary Table S2. The composition of olivine and orthopyroxene used for the calculation of the *f*O_2_ for SVT06 are from ref.^18^. The pressure (P) and temperature (T) used for the *f*O_2_ calculations on samples GSD07 and GSD08 are 3.5 GPa and 850 °C from ref.^19^, whereas P-T values for SVT06 of 5.5 GPa and 800 °C are from ref.^67^. For BDN02, P-T of 3 GPa and 800 °C are from ref.^14^ and are representative of the M3-1 and M3-2 metasomatic events. Given the majoritic nature of garnet in sample UGL03, pressure has been calculated using the geobarometer of ref.^68^ and temperature estimated using a lithospheric geotherm of 50.5 mW m^−2^. Oxygen fugacity values for BDN03 and UGL01 are from ref.^13^.

To model the *f*O_2_ of the unmetasomatised mantle end-member we started from the chemical composition in major oxides and calculated the composition of mineral phases in equilibrium at P ranging from 3 to 6 GPa along a 50 mW m^−2^ geotherm using THERMOCALC^69^ with the dataset of ref.^70^. Oxygen fugacity was calculated using the *GtfO2* software of ref.^21^. The 50 mW m^−2^ geotherm was chosen because it provides a good fit with the P-T trend of most samples; ~90% are within a ±100 °C range. We have estimated that the changes in *f*O_2_ at the pressures of interest, for ~100 °C temperature variation, are smaller than the uncertainty on *f*O_2_ calculated for unknown samples^21^. Because the thermodynamic dataset of ref.^70^ does not allow for calculation at pressures higher than 6 GPa, we have extrapolated the *f*O_2_ at higher pressure from lines of best fit to the lower pressure results. It has been shown that the bulk Fe_2_O_3_ content of garnet peridotites is directly related to their MgO wt% content^36^, so we used this relationship to calculate the Fe_2_O_3_ for each mantle end-member. We chose the dataset of ref.^36^ because it provides Fe_2_O_3_ of a wide range of mantle rock compositions, from dunite to websterite, measured on whole rock samples by Mössbauer spectroscopy. We used a regression line fitting these samples (R^2^ = 0.8) in a diagram of Fe^3+^/Fe_tot_ vs MgO wt% to calculate the Fe^3+^/Fe_tot_ content of each mantle end-member described in the text. To investigate the speciation of carbon in equilibrium with depleted mantle as function of *f*O_2_, we used the equilibrium between carbonate and graphite/diamond in a mantle assemblage expressed as:

$$\mathop{{{\rm{MgSiO}}}_{{\rm{3}}}}\limits_{{\rm{enstatite}}}+\mathop{{{\rm{MgCO}}}_{{\rm{3}}}}\limits_{{\rm{magnesite}}}=\mathop{{{\rm{Mg}}}_{{\rm{2}}}{{\rm{SiO}}}_{{\rm{4}}}}\limits_{{\rm{olivine}}}+{\rm{C}}+\,\mathop{{{\rm{O}}}_{{\rm{2}}}}\limits_{\mathrm{diamond}/\mathrm{graphite}}$$

This reaction is referred to by the acronym EMOD/G (enstatite-magnesite-olivine-diamond/graphite)^40^. In Fig. 3 the EMOD/G buffer is normalised to APM.

## Electronic supplementary material

Supplementary information
