# Supplementary material for: Garnet peridotites reveal spatial and temporal changes in the oxidation potential of subduction
Source: Sci Rep. 2018 Nov 6;8:16411. doi: 10.1038/s41598-018-34669-0 (PMC6219559; doi:10.1038/s41598-018-34669-0)
Supplement: Supplementary file 1 — Supplementary information [file 41598_2018_34669_MOESM1_ESM.pdf]

Garnet peridotites reveal spatial and temporal changes in the oxidation potential of subduction

Authors: Andrea Rielli[1*], Andrew G. Tomkins[2], Oliver Nebel[2], Joël Brugger[2], Barbara Etschmann[2] and David Paterson[3].

Affiliations

[1]Dipartimento di Scienze della Terra, Università di Pisa, Via S. Maria 53, 56126 Pisa, Italy.

[2]School of Earth, Atmosphere and the Environment, Monash University, Melbourne, Victoria 3800, Australia.

[3]Australian Synchrotron, 800 Blackburn Road, Clayton, Victoria 3168, Australia.

[*]Corresponding Author Details:

Email: andrea.rielli@dst.unipi.it

Phone: +39 05 03152276

Supplementary Figure S1

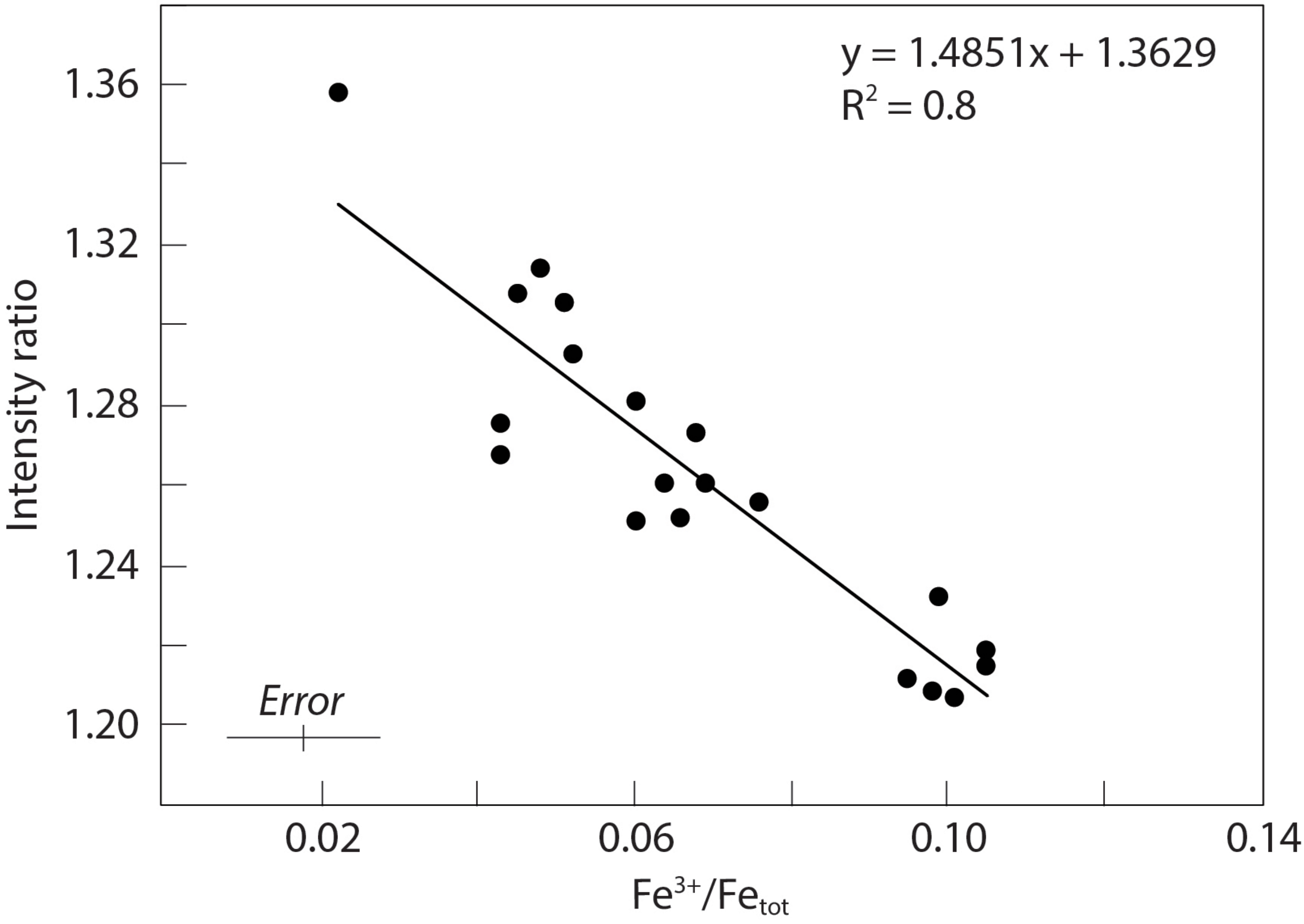

Supplementary Table S1

Standards

ID	7138.4 eV	7161.7 eV	Intensity ratio	Mössbauer Fe^{3+}/Fe_{tot}
**Dvk_1**	0.868	1.057	1.219	0.105
**Dvk_2**	0.789	1.071	1.358	0.022
**Dvk_3**	0.873	1.061	1.215	0.105
**Dvk_4**	0.871	1.093	1.256	0.076
**Dvk_6**	0.858	1.073	1.252	0.066
**Dvk_5**	0.871	1.072	1.232	0.099
**Dvk_7**	0.874	1.054	1.207	0.101
**Dvk_8**	0.891	1.077	1.208	0.098
**Dvk_10**	0.858	1.074	1.251	0.060
**LET6-2**	0.877	1.118	1.275	0.043
**LET-21**	0.836	1.093	1.308	0.045
**LET-7**	0.838	1.102	1.314	0.048
**LET-1**	0.864	1.095	1.267	0.043
**KIM44**	0.843	1.063	1.261	0.069
**KIM35**	0.862	1.105	1.281	0.060
**KIM30**	0.841	1.087	1.292	0.052
**LET9**	0.877	1.063	1.212	0.095
**KIM17**	0.850	1.071	1.261	0.064
**Kim39**	0.869	1.135	1.306	0.051
**Kim13**	0.876	1.115	1.273	0.068
**Kim1**	0.869	1.094	1.259	0.054

Samples

ID	Spectrum n°	7138.4 eV	7161.7 eV	Intensity ratio	Calculated Fe^{3+}/Fe_{tot}
**GSD07_1**	88	0.850	1.110	1.306	0.038
**GSD07_2**	89	0.830	1.090	1.313	0.033
**GSD07_3**	90	0.839	1.101	1.313	0.034
**GSD07_4**	91	0.845	1.088	1.287	0.051
**GSD07_5**	92	0.847	1.097	1.294	0.046
**GSD08_1**	100	0.830	1.080	1.301	0.042
**GSD08_2**	101	0.840	1.100	1.310	0.036
**GSD08_3**	102	0.840	1.090	1.298	0.044
**GSD08_4**	103	0.857	1.115	1.300	0.042
**GSD08_5**	104	0.829	1.082	1.306	0.039
**GSD08_6**	106	0.850	1.090	1.282	0.054
**BDN02_1**	110	0.840	1.110	1.321	0.028
**BDN02_2**	111	0.850	1.110	1.306	0.038
**BDN02_3**	112	0.850	1.120	1.318	0.030
**UGL03_2**	115	0.840	1.110	1.321	0.028
**UGL03_3**	116	0.850	1.080	1.271	0.062
**SVT06_1**	93	0.893	1.108	1.241	0.082
**SVT06_2**	94	0.877	1.072	1.223	0.094
**SVT06_3**	95	0.894	1.112	1.244	0.080
**SVT06_4**	96	0.892	1.098	1.231	0.089

Supplementary Table S2

	GSD07							
wt %	GSD07_1 (88) **Grt_1 core 1**	GSD07_2 (89) **Grt_1 core 2**	GSD07_3 (90) **Grt_1 rim 1**	GSD07_5 (91) **Grt _2 core**	GSD07_5 (92) **Grt _2 rim**	**Opx**	**Ol**	**Cpx**
**SiO_2**	41.99	42.06	42.17	42.16	41.93	57.80	41.45	54.66
**TiO_2**	0.14	0.10	0.11	0.10	0.08	0.00	0.00	0.19
**Al_2O_3**	20.85	20.86	21.15	21.02	20.63	0.90	0.00	3.18
**Cr_2O_3**	3.24	3.32	3.19	3.26	3.26	0.40	0.02	2.55
**Fe_2O_3**	0.34	0.30	0.29	0.45	0.45	n.d.	n.d.	n.d.
**FeO**	7.60	7.68	7.41	7.48	8.35	4.80	7.43	1.41
**MnO**	0.38	0.45	0.39	0.38	0.51	0.10	0.12	0.07
**MgO**	20.60	20.28	20.57	20.56	19.73	35.40	50.52	14.41
**CaO**	4.52	4.69	4.59	4.58	4.72	0.10	0.00	19.86
**Na_2O**	0.04	0.03	0.06	0.03	0.02	0.00	0.01	2.73
**NiO**	0.01	0.01	0.02	0.01	0.01	0.10	0.39	0.04
**K_2O**	0.01	0.01	0.00	0.01	0.00	0.00	0.01	0.01
**Total**	99.72	99.77	99.93	100.03	99.70	99.60	99.96	99.11
**Si**	3.01	3.01	3.01	3.01	3.02	1.98	1.01	1.99
**Ti**	0.01	0.01	0.01	0.01	0.00	0.00	0.00	0.01
**Al**	1.76	1.76	1.78	1.77	1.75	0.04	0.00	0.14
**Cr**	0.18	0.19	0.18	0.18	0.19	0.01	0.00	0.07
**Fe^{3+}**	0.02	0.01	0.01	0.02	0.02			0.00
**Fe^{2+}**	0.46	0.46	0.44	0.45	0.50	0.14	0.15	0.04
**Mn**	0.02	0.03	0.02	0.02	0.03	0.00	0.00	0.00
**Mg**	2.20	2.17	2.19	2.19	2.12	1.81	1.83	0.78
**Ca**	0.35	0.36	0.35	0.35	0.36	0.00	0.00	0.77
**Na**	0.01	0.00	0.01	0.00	0.00	0.00	0.00	0.19
**Ni**	0.00	0.00	0.00	0.00	0.00	0.00	0.01	0.00
**K**	0.00	0.00	0.00	0.00	0.00	0.00	0.00	0.00
**tot cat**	8.01	8.00	8.00	8.00	8.00	3.99	2.99	4.00
**Mg#**	82.86	82.48	83.18	83.05	80.81	92.93	92.38	94.80
**Fe^{3+}/Fe_{tot}**	0.038	0.033	0.034	0.051	0.046			

Supplementary Table S2 - continued

	GSD08; Grt-peridotite				
	GSD08_1 (101)	GSD08_2 (100)			
**wt %**	**Grt _1 core**	**Grt _1 rim**	**Opx**	**Ol**	**Cpx**
**SiO_2**	42.18	42.31	58.33	40.45	54.80
**TiO_2**	0.12	0.10	0.02	0.01	0.14
**Al_2O_3**	20.92	20.61	0.58	0.02	2.80
**Cr_2O_3**	2.25	2.63	0.15	0.01	2.28
**Fe_2O_3**	0.38	0.40	n.d.	n.d.	n.d.
**FeO**	9.06	8.23	5.24	7.62	1.66
**MnO**	0.52	0.45	0.11	0.06	0.03
**MgO**	19.80	20.03	35.32	50.49	14.66
**CaO**	4.43	4.53	0.13	0.01	20.57
**Na_2O**	0.03	0.05	0.02	0.00	2.44
**NiO**	0.00	0.02	0.08	0.41	0.04
**K_2O**	0.02	0.01	0.01	0.00	0.01
**Total**	99.71	99.36	99.99	99.08	99.40
**Si**	3.03	2.93	2.00	0.99	1.99
**Ti**	0.01	0.00	0.00	0.00	0.00
**Al**	1.77	1.90	0.02	0.00	0.12
**Cr**	0.13	0.15	0.00	0.00	0.07
**Fe^{3+}**	0.02	0.02	n.d.	n.d.	n.d.
**Fe^{2+}**	0.54	0.50	0.15	0.16	0.05
**Mn**	0.03	0.03	0.00	0.00	0.00
**Mg**	2.12	2.13	1.80	1.85	0.79
**Ca**	0.34	0.35	0.00	0.00	0.80
**Na**	0.00	0.00	0.00	0.00	0.17
**Ni**	0.00	0.00	0.00	0.01	0.00
**K**	0.00	0.00	0.00	0.00	0.00
**tot cat**	8.00	8.03	3.99	3.01	4.00
**Mg#**	79.57	80.92	92.31	92.10	94.04
**Fe^{3+}/Fe_{tot}**	0.042	0.036			

Supplementary Table S2 - continued

	**GSD08; Grt-pyroxenite vein**						
	GSD08_3 (103)	GSD08_4 (102)	GSD08_5 (104)	GSD08_6 (106)			
**wt %**	**Grt_2 core**	**Grt_2 rim**	**Grt_3 core**	**Grt_3 rim**	**Opx**	**Ol**	**Cpx**
**SiO_2**	40.62	41.52	41.52	41.19	58.34	41.34	55.22
**TiO_2**	0.03	0.03	0.05	0.04	0.04	0.02	0.13
**Al_2O_3**	23.64	23.09	23.41	23.22	0.66	0.01	2.95
**Cr_2O_3**	1.48	1.43	1.60	1.58	0.06	0.01	0.32
**Fe_2O_3**	0.41	0.45	0.38	0.55	n.d.		n.d.
**FeO**	8.31	8.76	8.42	8.64	4.81	7.35	1.50
**MnO**	0.48	0.57	0.42	0.55	0.07	0.07	0.04
**MgO**	20.55	19.77	20.66	19.86	35.64	50.40	15.44
**CaO**	4.30	4.53	4.41	4.39	0.11	0.01	21.88
**Na_2O**	0.01	0.01	0.02	0.01	0.01	0.01	1.89
**NiO**	0.00	0.02	0.04	0.01	0.08	0.37	0.04
**K_2O**	0.01	0.00	0.00	0.01	0.01	0.01	0.01
**Total**	99.83	100.17	100.93	100.04	99.83	99.60	99.41
**Si**	2.90	2.96	2.94	2.95	2.00	0.99	2.00
**Ti**	0.00	0.00	0.00	0.00	0.00	0.00	0.00
**Al**	1.99	1.94	1.95	1.96	0.03	0.00	0.13
**Cr**	0.08	0.08	0.08	0.08	0.00	0.00	0.01
**Fe^{3+}**	0.02	0.02	0.02	0.03	0.00		0.00
**Fe^{2+}**	0.52	0.52	0.50	0.52	0.14	0.16	0.05
**Mn**	0.03	0.04	0.03	0.03	0.00	0.00	0.00
**Mg**	2.19	2.10	2.18	2.12	1.82	1.85	0.83
**Ca**	0.33	0.34	0.33	0.34	0.00	0.00	0.85
**Na**	0.00	0.00	0.00	0.00	0.00	0.00	0.13
**Ni**	0.00	0.00	0.00	0.00	0.00	0.01	0.00
**K**	0.00	0.00	0.00	0.00	0.00	0.00	0.00
**tot cat**	8.05	8.02	8.04	8.02	3.99	3.01	4.00
**Mg#**	80.93	80.04	81.39	80.38	92.23	92.26	94.83
**Fe^{3+}/Fe_{tot}**	0.044	0.042	0.039	0.054			

Supplementary Table S2 - continued

	SVT06				
	SV06_1 (93)	SV06_2 (94)	SV06_3 (95)	SV06_4 (96)	
**wt %**	**Grt_1**	**Grt_2**	**Gr_ 3**	**Grt_4**	**Cpx**
**SiO_2**	40.35	40.14	40.27	40.36	53.62
**TiO_2**	0.07	0.07	0.07	0.04	0.05
**Al_2O_3**	22.41	22.27	22.34	22.16	1.31
**Cr_2O_3**	0.08	0.10	0.12	0.19	0.07
**Fe_2O_3**	1.58	1.85	1.51	1.70	n.d.
**FeO**	15.88	16.01	15.67	15.70	4.23
**MnO**	0.86	0.90	0.81	0.90	0.16
**MgO**	14.45	14.12	14.19	14.35	15.49
**CaO**	4.70	4.60	4.99	4.44	23.00
**Na_2O**	0.01	0.04	0.02	0.01	0.57
**NiO**	0.02	0.02	0.02	0.02	0.02
**K_2O**	0.02	0.02	0.01	0.00	0.04
**Total**	100.43	100.13	100.00	99.89	98.57
**Si**	2.98	2.97	2.98	2.99	1.99
**Ti**	0.00	0.00	0.00	0.00	0.00
**Al**	1.95	1.94	1.95	1.93	0.06
**Cr**	0.00	0.01	0.01	0.01	0.00
**Fe^{3+}**	0.08	0.09	0.08	0.09	0.00
**Fe^{2+}**	0.98	0.99	0.97	0.97	0.13
**Mn**	0.05	0.06	0.05	0.06	0.01
**Mg**	1.59	1.56	1.57	1.58	0.86
**Ca**	0.37	0.36	0.40	0.35	0.91
**Na**	0.00	0.01	0.00	0.00	0.04
**Ni**	0.00	0.00	0.00	0.00	0.00
**K**	0.00	0.00	0.00	0.00	0.00
**tot cat**	8.01	8.00	8.00	7.99	4.00
**Mg#**	61.86	61.11	61.74	61.97	86.72
**Fe^{3+}/Fe_{tot}**	0.082	0.094	0.080	0.089	

Supplementary Table S2 - continued

	BDN02				
	BDN02_1 (110)	BDN02_2 (111)	BDN02_3 (112)		
**wt %**	**Grt corona**	**Grt exsolution**	**Grt vein**	**Ol**	**opx**
**SiO_2**	41.97	41.66	40.87	40.22	57.90
**TiO_2**	0.03	0.02	0.02	0.00	0.01
**Al_2O_3**	21.33	21.47	21.59	0.38	1.32
**Cr_2O_3**	4.37	4.24	4.34	0.00	0.30
**Fe_2O_3**	0.30	0.27	0.36	n.d.	
**FeO**	8.56	8.30	8.16	6.95	4.68
**MnO**	0.53	0.53	0.52	0.13	0.14
**MgO**	19.47	19.50	19.62	50.58	35.58
**CaO**	5.76	5.51	5.29	0.00	0.19
**Na_2O**	0.03	0.03	0.03	0.00	0.02
**NiO**	0.01	0.00	0.01	0.51	0.09
**K_2O**	0.01	0.00	0.01	0.00	0.01
**Total**	102.37	101.53	100.84	98.76	100.23
**Si**	2.96	2.96	2.93	0.99	1.98
**Ti**	0.00	0.00	0.00	0.00	0.00
**Al**	1.77	1.80	1.82	0.01	0.05
**Cr**	0.24	0.24	0.25	0.00	0.01
**Fe^{3+}**	0.01	0.01	0.01	0.00	0.00
**Fe^{2+}**	0.51	0.50	0.49	0.14	0.13
**Mn**	0.03	0.03	0.03	0.00	0.00
**Mg**	2.05	2.06	2.09	1.85	1.81
**Ca**	0.44	0.42	0.41	0.00	0.01
**Na**	0.00	0.00	0.00	0.00	0.00
**Ni**	0.00	0.00	0.00	0.01	0.00
**K**	0.00	0.00	0.00	0.00	0.00
**tot cat**	8.02	8.02	8.04	3.01	3.99
**Mg#**	80.02	80.61	80.95	92.84	93.13
**Fe^{3+}/Fe_{tot}**	0.028	0.030	0.038		

Supplementary Table S2 - continued

	UGL03				
	UGL03_1 (114)	UGL03_2 (115)	UGL03_3 (116)		
**wt %**	**Grt_veinlet core**	**Grt_vein core 1**	**Grt_vein core 2**	**cpx**	**opx**
**SiO_2**	42.66	42.50	42.65	55.01	58.93
**TiO_2**	0.03	0.04	0.01	0.05	0.02
**Al_2O_3**	20.62	20.76	20.47	2.67	0.51
**Cr_2O_3**	2.92	2.94	2.87	2.19	0.18
**Fe_2O_3**	0.05	0.26	0.57	n.d.	
**FeO**	7.73	8.06	7.78	1.01	4.17
**MnO**	0.36	0.36	0.41	0.06	0.06
**MgO**	20.54	20.43	20.81	15.21	35.97
**CaO**	4.57	4.69	4.20	21.30	0.16
**Na_2O**	0.03	0.00	0.02	1.92	0.02
**NiO**	0.01	0.01	0.02	0.06	0.14
**K_2O**	0.01	0.01	0.01	0.01	0.01
**Total**	99.53	100.01	99.69	99.47	100.17
**Si**	3.05	3.03	3.05	1.99	2.00
**Ti**	0.00	0.00	0.00	0.00	0.00
**Al**	1.74	1.75	1.72	0.11	0.02
**Cr**	0.17	0.17	0.16	0.06	0.00
**Fe^{3+}**	0.00	0.01	0.03	0.00	0.00
**Fe^{2+}**	0.46	0.48	0.47	0.03	0.12
**Mn**	0.02	0.02	0.02	0.00	0.00
**Mg**	2.19	2.17	2.22	0.82	1.82
**Ca**	0.35	0.36	0.32	0.83	0.01
**Na**	0.00	0.00	0.00	0.13	0.00
**Ni**	0.00	0.00	0.00	0.00	0.00
**K**	0.00	0.00	0.00	0.00	0.00
**tot cat**	7.99	8.00	8.00	3.99	3.98
**Mg#**	82.57	81.88	82.66	96.42	93.89
**Fe^{3+}/Fe_{tot}**	0.017	0.028	0.062		
